# Wen-pi-tang-Hab-Wu-ling-san, a Polyherbal Medicine, Attenuates ER Stress in 3T3-L1 Preadipocytes by Promoting the Insulin Signaling Pathway

**DOI:** 10.1155/2013/825814

**Published:** 2013-12-22

**Authors:** Yunkyung Han, Hyo Won Jung, Hyo Sang Bae, Yong-Ki Park

**Affiliations:** ^1^Oriental Medicine R&D Center, Dongguk University, Gyeongju 780-714, Republic of Korea; ^2^Department of Herbology, College of Oriental Medicine, Dongguk University, Gyeongju 780-714, Republic of Korea; ^3^Department of Sasang Constitutional Medicine, Dongguk University Ilsan Oriental Hospital, Dongguk University, Gyeonggi-Do 410-773, Republic of Korea

## Abstract

The endoplasmic reticulum (ER) is an organelle that functions to synthesize, fold, and transport proteins. ER stress is a key link between type 2 diabetes (T2D), obesity, and insulin resistance. In this study, we investigated the effect of WHW on the ER stress response and the insulin signaling pathway in 3T3-L1 preadipocytes. 3T3-L1 preadipocytes were differentiated into adipocytes, and ER stress was then induced by treatment with tunicamycin. ER stress-induced adipocytes were treated with different concentrations of WHW for 24 h. The expression of ER stress-related molecules such as X-box-binding protein-1 (XBP-1), glucose-regulated protein 78 (GRP78), C/EBP-homologous protein 10 (CHOP10), and eukaryotic initiation factor 2**α** (eIF2**α**) and signaling molecules such as phosphatidylinositol 3-kinase (PI3K), insulin receptor substrates-1 (IRS-1), and c-Jun N-terminal protein kinase (JNK) were investigated. WHW significantly inhibited the expression of XBP-1, GRP78, CHOP10, and eIF2**α** in ER stress-induced 3T3-L1 adipocytes. WHW also increased the PI3K expression and the IRS-1 phosphorylation but decreased the phosphorylation of JNK in ER stress-induced 3T3-L1 adipocytes. Our results indicate that WHW inhibits ER stress in adipocytes by suppressing the expression of ER stress-mediated molecules and the insulin signaling pathway, suggesting that WHW may be an attractive therapeutic agent for managing T2D.

## 1. Introduction

Obesity is the leading risk factor for the development of many life-threatening diseases, particularly insulin resistance and type 2 diabetes (T2D) [1]. Although the mechanisms by which obesity contributes to insulin resistance and T2D remain the subject of intensive investigation, recent studies suggest that endoplasmic reticulum (ER) stress plays a major role in mediating obesity-induced insulin resistance and T2D [2].

The ER is a critical intracellular organelle that coordinates the synthesis, folding, and transport of proteins [3, 4]. A variety of biochemical or pathophysiological stimuli can interrupt the protein folding process in the ER by disrupting protein glycosylation, disulfide bond formation, or the ER calcium pool. These disruptions can cause the accumulation of unfolded or misfolded proteins in the ER lumen, a condition termed as ER stress [5]. The presence of unfolded proteins in the ER is sensed, and activation of the unfolded protein response (UPR) is regulated by ER chaperones and folding enzymes, such as glucose-regulated protein 78 (GRP78), X-box-binding protein-1 (XBP-1), C/EBP-homologous protein 10 (CHOP10), and eukaryotic initiation factor2*α* (eIF2*α*) [6, 7]. Under physiological conditions, these chaperones and folding enzymes are constitutively expressed [6].

T2D is characterized by insulin resistance [8]. Normally, activated insulin receptors phosphorylate proximal signaling molecules, such as insulin receptor substrate 1 (IRS-1), which transduces the effects of insulin through interaction with cytosolic targets [9]. The phosphorylation of IRS-1 subsequently stimulates phosphatidylinositol 3-kinase (PI3K), which is an important step for stimulating insulin-induced glucose transport [10]. In obesity, tyrosine phosphorylation of IRS-1 is inhibited by c-Jun N-terminal protein kinase (JNK)-dependent serine phosphorylation of IRS-1 [9]. Although the underlying mechanisms are not yet fully understood, it has been reported that the mechanism of obesity-related JNK activation plays a critical role in ER stress-induced insulin resistance [2, 9].

Wen-pi-tang-Hab-Wu-ling-san (WHW) is a polyherbal medicine originating from oriental prescriptions for treatment of renal diseases including chronic renal failure (CRF) and diabetic nephropathy [11, 12]. Recently, WHW was studied for its multiple pharmaceutical properties such as anti-kidney fibrosis effects in ischemia/reperfusion and ureteral obstruction-induced renal injuries in mice [11, 13], TGF-beta-induced epithelial-mesenchymal transdifferentiation in kidney cells [14], anti-inflammatory [11, 15], antioxidative [11], and kidney protection effects by induction of heat shock protein [13], as well as recently reported antidiabetic nephropathy in rats [12]. However, WHW and its antidiabetic mechanism have not yet been investigated.

Therefore, we investigated the effect of WHW on the ER stress response through the insulin signaling pathway in 3T3-L1 adipocytes. Our study provides evidence of an effect of WHW on ER stress-induced insulin resistance and clarifies its action mechanism in obesity-induced insulin resistance and T2D.

## 2. Materials and Methods

WHW extract was prepared from 14 herbs as described previously [16]. The constituents of WHW were purchased from Medicinal Materials Company (Youngcheon province) and authenticated by Professor Yong-Ki Park, a medicinal botanist. Voucher specimens (OB05-1) have been deposited in the herbarium of Oriental Medicine R&D Center, Dongguk University, Republic of Korea. WHW was received from Hanpoong Pharm and Food Co., Ltd. (Jeonju, Republic of Korea). Briefly, herbs of WHW (50 kg) were mixed according to constitution ratio^12^, minced with a grinder, extracted with 1000 mL of boiling water (98°C) for 3 h, and filtered with 50 *μ*m and 1 *μ*m cartridge papers. The filtered extract was concentrated at 55°C, 700 mmHg for 15 hr, and the concentrated extract was vacuum-dried (yield: 23.7~25%).

3T3-L1 preadipocytes (ATCC, Manassas, VA, USA) were cultured in high-glucose Dulbecco's Modified Eagle's Medium (DMEM) supplemented with 10% bovine calf serum (BCS; Hyclone, Logan, UT, USA) at 37°C under a 5% CO_2_ atmosphere. Two days after confluence, the cells were induced to differentiate with adipogenic agents (0.5 mM 3-isobutyl-1-methylxanthine (IBMX), 1 *μ*M dexamethasone, and 5 *μ*g/mL insulin) in DMEM containing 10% fetal bovine serum (FBS; Hyclone). After 48 h, cells were maintained in postdifferentiation medium containing 10% FBS and 5 *μ*g/mL insulin for 1 additional day. The cells were switched to fresh postdifferentiation media every 2 days for 6 days.

To induce ER stress, cells were supplemented with fresh medium containing 2 *μ*g/mL tunicamycin (Sigma Aldrich, St. Louis, MO, USA). After 24 h, the cells were treated with appropriate concentrations of WHW extract. WHW extracts were dissolved in postdifferentiation media.

Total RNA was purified from 3T3-L1 adipocytes according to a protocol that was described previously [17]. The cDNA was generated from 5 *μ*g of total RNA. The RT reaction was performed in a reaction mixture (Promega, Madison, WI, USA). The PCR products were electrophoresed in 1% agarose gels at 100 V and verified by assessing their predicted sizes under UV light. Oligonucleotide primer sequences were as follows: XBP-1 (accession no. NM 013842.2) Fw: 5′-AAA CAG AGT AGC AGC GCA GAC TGC-3′ and Rv: 5′-GGA TCT CTA AAA CTA GAG GCT TGG TG-3′; GRP78 (accession no. NM 022310.3) Fw: 5′-ACC TAT TCC TGC GTC GGT GT-3′ and Rv: 5′-GCA TCG AAG ACC GTG TTC TC-3′; and GAPDH (accession no. XM 994067.2) Fw: 5′-CTC CTG GAG TCT ACT GGT GT-3′ and Rv: 5′-GTC ATC ATA CTT GGC AGG TT-3′. GAPDH was used as an internal control for PCR.

For western blot assay, 3T3-L1 cells were added to Lysis buffer (10 mM Tris-HCl, pH 7.9, 10 mM NaCl, 3 mM MgCl_2_, and 1% NP-40) and detached with a scraper. 30 *μ*g of total protein was separated on SDS-PAGE gels and transferred onto nitrocellulose membranes. Incubation with primary and secondary antibodies was either overnight at 4°C or at room temperature for 1 h. The antibodies used in this study were anti-IRS-1 (1 : 1000, Cell Signaling Technology, Beverly, MA, USA), anti-phospho-IRS-1 (1 : 1000, Cell Signaling), anti-PI3 K (1 : 1000, Cell Signaling), anti-JNK (1 : 1000, Cell Signaling), anti-phospho-JNK (1 : 500, Santa Cruz Biotechnology, Santa Cruz, CA, USA), anti-*β*-actin (1 : 1000, Sigma Aldrich), and HRP-labeled anti-rabbit or mouse IgG (1 : 5000; Santa Cruz Biotechnology).

Quantitative data from all experiments are expressed as the mean ± SD and are representative of three independent experiments. Statistical analysis was carried out by one-way ANOVA with the post hoc test using GraphPad Prism 5.0 statistical analysis software (GraphPad Software, Inc., San Diego, CA, USA). Values of *P* < 0.05 were considered significant.

## 3. Results and Discussion

**Figure 1:**
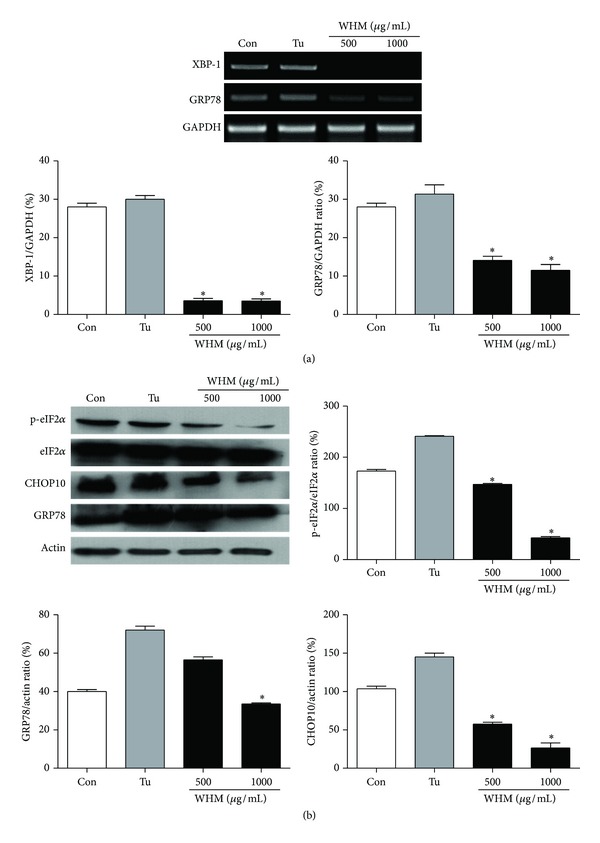
Effect of WHW extract on tunicamycin-induced ER stress indicators in 3T3-L1 cells. The cells were treated with WHW extract at concentrations of 500 and 1000 *μ*g/mL for 9 days. (a) The expressions of XBP-1, GRP 78, and GAPDH mRNA were analyzed by RT-PCR. GAPDH was used as the internal control. (b) The expression of eIF2*α*, CHOP10, or GRP78 was measured by western blot analysis. Relative density was calculated as the ratio of p-eIF2*α* expression to eIF2*α* expression or of actin expression to expressions of CHOP10 or GRP78 expression, respectively. Con: differentiated adipocytes as a control; Tu: 2 *μ*g/mL tunicamycin; 500: 2 *μ*g/mL tunicamycin + WHW 500 *μ*g/mL; 1000: 2 *μ*g/mL tunicamycin + WHW 1000 *μ*g/mL. These data are presented as the mean ± SD (*n* = 3). *Significantly different (*P* < 0.05) from tunicamycin treatment.

ER stress has been recognized as an important mechanism for obesity-related T2D and insulin resistance [2]. In this study, we focused on the effect of WHW on ER stress and the insulin signaling pathway in 3T3-L1 adipocytes. In our study, WHW had antidiabetic activity by inhibiting the expression of ER stress-mediated molecules and upregulating the sensitivity in T2D. Tunicamycin is a commonly used agent known to induce ER stress by inhibiting *N*-linked glycosylation of luminal ER proteins [2, 18]. In this study, exposure of adipocytes to 2 *μ*g/mL tunicamycin caused an increase in the expression of ER stress markers such as XBP-1, GRP78, CHOP10, and eIF2*α* (Figure 1). On the other hand, mRNA levels of XBP-1 and GRP78 were significantly downregulated by WHW treatment in 3T3-L1 adipocytes (Figure 1(a)). Moreover, the expression of CHOP10 and phospho-eIF2*α* was also significantly reduced with WHW treatment (Figure 1(b)). These results suggest that exposure of adipocytes to WHW can regulate the key processes of ER stress like phosphorylation of eIF2*α*, CHOP10, and XBP-1 and the downregulation of GRP78.

**Figure 2:**
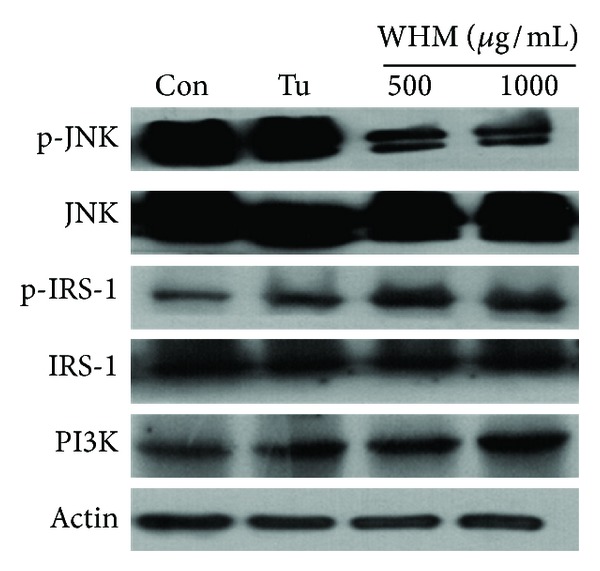
Effect of WHW extract on insulin signaling pathway in 3T3-L1 cells. The cells were treated with WHW extract at concentrations of 500 and 1000 *μ*g/mL for 9 days. The expression of JNK, IRS-1, or PI3K was measured by western blot analysis. Relative density was calculated as the ratio of p-JNK expression to JNK, p-IRS-1 expression to IRS-1 expression, or PI3K expression to actin expression, respectively. Con: differentiated adipocytes as a control; Tu: 2 *μ*g/mL tunicamycin; 500: 2 *μ*g/mL tunicamycin + WHW 500 *μ*g/mL; 1000: 2 *μ*g/mL tunicamycin + WHW 1000 *μ*g/mL. These data are presented as the mean ± SD (*n* = 3). *Significantly different (*P* < 0.05) from tunicamycin treatment.

An important characteristic of T2D is insulin resistance. It has been reported that the ER stress response in insulin resistance occurs through activated JNK, which participates in insulin resistance by inhibiting phosphorylation of IRS-1, leading to impaired insulin signaling [19]. Therefore, we investigated the effect of WHW on insulin signaling pathway under ER stress. We found that the phosphorylation of JNK was significantly inhibited, while the expression of IRS-1 and PI3K was significantly increased by WHW treatment (Figure 2). These results indicate that WHW may improve ER stress, which induces insulin resistance by activating the insulin signaling pathway.

## 4. Conclusions

In summary, we have demonstrated that WHW strongly inhibits the expression of ER stress markers, such as XBP-1, GRP78, CHOP10, and eIF2*α*. Furthermore, this action mechanism of WHW underlies the improvement in the ER stress-induced impairment of insulin signaling molecules. Based on these findings, we suggest that WHW can prevent ER stress in the development of insulin resistance in T2D and could be used as a potential therapy for T2D and T2D-related complications like diabetic nephropathy.
